# Digital literacy and positive psychological functioning among college students in the digital era: evidence from a multi-university sample in Henan Province, China

**DOI:** 10.3389/fpsyg.2026.1966399

**Published:** 2026-10-29

**Authors:** Zihan Yang, Na Li, Hao Li

**Affiliations:** 1 School of Business, Xinyang Normal University, Xinyang, China; 2 Enrollment and Employment Office of Xinyang Normal University, Xinyang, China

**Keywords:** college students, digital era, digital literacy, digital tool use, positive psychological functioning

## Abstract

**Introduction:**

This study examines the association between digital literacy and positive psychological functioning among college students, and explores dimension-specific patterns, heterogeneity, and exploratory associational decomposition.

**Methods:**

Data were obtained from a cross-sectional survey of 893 students from 18 universities in Henan Province, China, conducted in 2024. Digital literacy was operationalized across five dimensions: digital tool use proficiency, information acquisition and processing, digital social interaction, digital innovation, and digital security and ethical awareness. Positive psychological functioning was assessed across six dimensions of psychological functioning and adaptive adjustment. Ordinary least squares models, alternative index-construction methods, wild-cluster bootstrap inference, multiplicity-adjusted exploratory analyses, continuous-variable interaction and non-linearity tests, and a supplementary instrumental-variable-based robustness check were employed.

**Results:**

Higher overall digital literacy was positively associated with positive psychological functioning. In the adjusted model, a 10-point increase on the rescaled 0–100 digital-literacy index corresponded to approximately a 1.78-point higher score on the 0–100 positive psychological functioning composite, while the model explained a modest proportion of outcome variance. After small-cluster and multiplicity corrections, information acquisition and processing remained negatively associated with positive psychological functioning, whereas digital social interaction remained positively associated; digital tool use proficiency, digital innovation, and digital security and ethical awareness were not robustly associated with the outcome. Exploratory heterogeneity and non-linearity analyses provided no robust evidence of moderation or non-linear functional form after conservative correction, although the living-expense interaction showed suggestive evidence. Exploratory decomposition analyses identified statistically detectable indirect components through digital information regulation behavior, online social boundary practice, and structured digital use practice.

**Discussion:**

The findings support a cautious, multidimensional interpretation of digital literacy as one correlate of students' psychological functioning rather than as a standalone psychological-intervention.

## 1 Introduction

### 1.1 Research background

The profound integration of digital technologies is reshaping human life, particularly driving generational shifts in higher education. In China, internet penetration reached 78.6% by the end of 2024, while the 2025 White Paper on Smart Education emphasized the expansion of digital infrastructure, high-quality educational resources, and large-scale digital applications (China Internet Network Information Center, 2025; Ministry of Education of the People's Republic of China, 2025). These developments have reshaped how students learn, communicate, access services, and organize everyday life. At the same time, continuous connectivity may expose students to information overload, social comparison, privacy risks, and problematic patterns of digital use. These parallel opportunities and risks make college students an important population for examining the relationship between digital literacy and positive psychological functioning (Zhao et al., 2024).

Digital literacy refers to individuals' abilities to effectively acquire, evaluate, use, and create digital information in digital settings, as well as their understanding, application and management of digital technologies. It is an essential skill for college students in the digital-intelligent era (Chetty et al., 2018). College students with stronger digital literacy may be better equipped to address digital-technology-related challenges, make effective use of digital resources, and identify potentially harmful or misleading information. These capacities may be associated with better psychological functioning by reducing information-related stress and supporting more purposeful digital engagement. Moreover, stronger digital literacy may support more purposeful management of time spent on digital devices and online platforms and may facilitate healthier patterns of digital engagement (Choi, 2020). In this study, the “digital-intelligent era” refers to a stage in which networked digital technologies and intelligent systems are embedded in routine learning, social interaction, and decision-making. This digitally driven transformation creates new opportunities for access to information and social support, but it also exposes students to information overload, continuous connectivity, social comparison, privacy risks, and pressure to update digital skills.

Accordingly, the primary objective of this study is to examine whether digital literacy is associated with positive psychological functioning among college students in Henan Province. The study addresses three related questions: (1) Is overall digital literacy associated with students' positive psychological functioning? (2) Do the associations differ across digital-literacy dimensions, positive-psychological-functioning dimensions, and student characteristics? and (3) To what extent do digital information regulation behavior, online social boundary practice, and structured digital use practice statistically account for the observed cross-sectional association, while recognizing their conceptual overlap with digital literacy? The multidimensional indices are used as measurement tools to address these substantive questions rather than being treated as the principal contribution of the study.

This study contributes to the literature in three ways. First, it evaluates digital literacy and positive psychological functioning as multidimensional constructs in a relatively large sample of college students from different types of universities in Henan Province. Second, it jointly examines the overall association and dimension-specific patterns as primary contributions, with heterogeneity and associational decomposition reported as secondary exploratory analyses across demographic and educational groups, thereby identifying both potentially beneficial and potentially burdensome aspects of digital engagement. Third, it assesses three theoretically specified decomposition components and explicitly discusses the conceptual overlap between broad digital literacy and its behavioral manifestations. These contributions are empirical and measurement-oriented; the study does not claim to establish a new general theory or definitive causal effects.

### 1.2 Research review

Digital literacy and college students' positive psychological functioning have attracted increasing scholarly attention. Existing studies have examined the conceptualization, determinants, and consequences of both constructs from multiple theoretical and empirical perspectives.

Digital literacy was first defined as the ability to understand and use digital technology to access information (Gilster, 1997). Early research focused on its definition, connotation, and basic components. As information technology evolved, the research perspective diversified. Digital literacy expanded to a multi-dimensional ability, including technology operation, information literacy, and media literacy. Studies began to focus on the “digital divide” caused by differences in technology mastery (Chetty et al., 2018). With the rapid development of emerging technologies like artificial intelligence and big data, new requirements for digital literacy emerged. Research shifted to adapting to technological changes, reshaping the connotation of digital literacy, and paying attention to its social impact and ways to enhance education. It also gradually focused on social equity and vulnerable groups (Eden et al., 2024). In the digital economy era, research emphasizes the comprehensiveness and adaptability of digital literacy for all citizens. Through education, training, and policy-making, it aims to improve the ability of different groups to respond to the challenges associated with increasingly intelligent and data-driven environments. For example, studies have begun to focus on “farmers' digital literacy” and “digital exclusion among older adults” (Mokhtar et al., 2022; Quan-Haase et al., 2018). Recent evidence among final-year university students in Indonesia showed that digital literacy, encompassing technical, cognitive, and socio-emotional competencies, was positively associated with digital satisfaction, digital wellness, and safe and responsible digital behavior, underscoring the broader relevance of multidimensional digital competence to students' digital wellbeing (Herdian et al., 2026).

Current research primarily centers on the types, causes, and intervention strategies for college students' psychological adjustment problems. Anxiety and depressive disorders are among the most common mental health conditions worldwide. College students may face additional academic, interpersonal, financial, and transition-related stressors that can affect their psychological wellbeing. At the individual level, physiological factors (e.g., genetic diseases, sleep disorders) and psychological traits (such as introversion, cognitive biases, and low stress-resistance) together create internal risks. Research indicates that physiological dysfunction can easily trigger emotional fluctuations, while negative cognitive patterns can exacerbate psychological imbalances (Shi et al., 2026). Family environment, parenting styles, and economic conditions significantly impact psychological functioning. A harmonious family helps foster a positive personality, whereas a conflict-filled family can lead to a lack of security. Overly protective or harsh parenting inhibits the development of psychological resilience, and economic pressure may induce feelings of inferiority and anxiety (Maglica et al., 2021). Schools also play a dual-edged role. Exam-oriented education intensifies academic pressure, while quality education promotes all-round development. Positive teacher-student interaction and a healthy campus culture can strengthen students' sense of belonging; the lack of these can cause psychological distress (Kondirolli and Sunder, 2022). At the social level, cultural conflict, competitive pressure, and the availability of social support constitute important external influences on students' psychological functioning. Materialism and other negative values can impact students' cognition, job competition can intensify anxiety, and insufficient social support can magnify psychological crises (Fasihi Harandi et al., 2017). In intervention practices, cognitive-behavioral therapy and psychological counseling are the main approaches. The academic community emphasizes that interventions should be based on China's educational reality. Intervention effectiveness can be enhanced through curriculum reform, optimization of consultation systems, and mental health promotion, forming culturally adaptive solutions. Currently, digital technologies like the internet are deeply integrated into the lifestyle of the youth, influencing their employment income, educational equity, satisfaction, and sense of gain, and profoundly affecting their social behavior and psychological development (Jameel et al., 2025). In studies on the impact of digital technologies such as the internet or mobile phones on the psychological functioning of college students, it has been pointed out that while digital technologies are developing rapidly, they are also creating digital inequality problems, such as the digital divide. Digitally advantaged groups can use online resources to expand access to information, social networks, educational opportunities, and employment resources. By contrast, students with limited digital skills may encounter greater difficulty navigating increasingly digital learning and social environments, which may be associated with poorer psychological adjustment (Chetty et al., 2018).

Studies have shown that college students with higher levels of digital literacy are better able to utilize online resources to access health-related information, which in turn has a positive impact on their psychological functioning (Li and Yang, 2024). Digital literacy also helps college students build good interpersonal relationships in online social interactions, which can alleviate feelings of loneliness and anxiety. In contrast, some studies have focused on college students with lower digital literacy. Due to their lack of ability to discern online information, these students are more susceptible to the influence of harmful online content and are prone to excessive addiction to the virtual online world, which can trigger psychological issues such as anxiety and depression (Batra et al., 2024).

Existing research provides important evidence on digital skills, online behavior, and student psychological functioning, but three gaps remain. First, many studies use a single indicator of internet use or health-related digital literacy and therefore do not distinguish between practical tool use, information processing, online social interaction, innovation, and security-related awareness. Second, prior research has focused mainly on average associations, with less attention to whether different dimensions are related to positive psychological functioning in different directions or whether the associations vary across student groups. Third, potential decomposition components are often discussed conceptually but are not clearly separated from the broader digital-literacy construct or formally evaluated with indirect-effect tests. The present study addresses these gaps by using multidimensional measures, examining dimension-specific and subgroup patterns, and testing three theoretically defined decomposition components while interpreting the results in light of the limitations of cross-sectional self-report data.

## 2 Theoretical foundations

### 2.1 Conceptualization and dimensions of digital literacy

Digital literacy is a complex ability system that continues to evolve with technological innovation. Its connotation has transcended the basic operation of tools and has developed into a comprehensive ability that integrates information processing, digital interaction, innovative practice, and ethical awareness (Norman and Skinner, 2006). This ability emphasizes the literacy requirements for efficient learning, collaboration, and innovation in the digital environment. It specifically includes five core dimensions:

(1) Digital Tool Use Proficiency: This covers the operation of smart devices and the use of professional software. For example, it involves the efficient integration of research data through literature management tools and the completion of cross-regional collaboration via online learning platforms.

(2) Information Acquisition and Processing: In response to the problem of information overload, it requires the mastery of precise retrieval techniques such as Boolean logic and the application of critical thinking to authenticate and evaluate the value of information, establishing a defense mechanism against information pollution.

(3) Digital Social Interaction: This includes online communication norms and collaborative practices. For example, it involves cross-cultural expression in social media and remote project management in virtual teams. It focuses on cultivating awareness of online etiquette and the ability to manage digital identities.

(4) Digital Innovation: Focusing on the creative transformation empowered by technology, it is manifested in programming development, digital art creation, and the design of solutions based on artificial intelligence. It emphasizes the integration and innovation of technological tools with professional knowledge.

(5) Digital Security and Ethical Awareness: This constructs a three-fold protection system that includes the application of data encryption technology, the recognition of legal boundaries of online behavior, and awareness of intellectual property rights protection. It forms a code of conduct for responsible digital citizens.

The 12-item digital-literacy instrument was developed as a study-specific operationalization rather than being adopted item-for-item from a single established psychometric scale. Conceptually, the five domains were organized around widely recognized components of digital competence: operational and problem-solving skills, information retrieval/evaluation/integration, digital communication and collaboration, digital content creation and innovation, and safe, ethical and responsible use. We first distinguished five primary dimensions, then decomposed each into secondary and tertiary sub-indicators, and finally translated each sub-indicator into a concrete behavioral manifestation that college students can recognize in daily life. This procedure ensures that every abstract competency is anchored to an observable behavior, which is essential for self-report measurement validity. The resulting 12-item scale is the independent-variable instrument used throughout the paper. All items were rated on a five-point Likert scale ranging from 1 (“strongly disagree”) to 5 (“strongly agree”), with higher scores indicating stronger digital literacy. The specific content is shown in Table 1.

**Table 1:** Comprehensive evaluation index system of college students' digital literacy.

Primary indicator	Secondary indicator	Tertiary indicator	Specific manifestation
Digital tool use proficiency	Operational proficiency	Software operation proficiency	Ability to independently complete computer-related technical operations
	Digital problem-solving	Practical problem-solving ability	Solving specific problems in learning/life through digital tools
Information acquisition and processing	Information retrieval capability	Keyword optimization ability	Using precise keywords to retrieve literature in academic databases
	Information assessment capability	Information discrimination ability	Ability to identify online rumors and false advertisements
	Information integration capability	Multi-source data integration ability	Integrating data from different sources (such as literature, charts) into a structured report
Digital social interaction	Online collaboration capability	Collaboration tool usage ability	Completing group tasks with the help of online office software
	Social etiquette and norms	Adherence to online etiquette	Respecting others' opinions and avoiding verbal violence in social media
	Risk response capability	Social risk prevention awareness	Regularly checking privacy settings and identifying online fraud behaviors
Digital innovation	Creative expression capability	Digital content creation ability	Producing original works such as short videos, programming development, etc.
	Knowledge integration capability	Cross-domain knowledge integration	Combining digital technology with professional field knowledge
Digital security and ethical awareness	Privacy protection awareness	Personal information management ability	Avoiding the disclosure of personal key information in public networks
	Technical ethical cognition	Data use ethical judgment ability	Complying with data collection authorization rules (e.g., not reprinting others' content without permission)

The five-domain structure was not copied from a single existing scale. Rather, it was synthesized from established digital-competence traditions. Digital Tool Use Proficiency draws on the operational/problem-solving dimension of digital competence (Chetty et al., 2018); Information Acquisition and Processing draws on information and data literacy frameworks (Norman and Skinner, 2006; Choi, 2020); Digital Social Interaction draws on communication and collaboration competence (Batra et al., 2024; Marchenko and Murzina, 2022). Digital Innovation draws on digital content creation and creative production (George et al., 2024; Tarifa-Rodriguez et al., 2025) and Digital Security and Ethical Awareness draws on safety, ethical, and responsible-use traditions (Ramos-Soler et al., 2018). Each domain was then contextualized for Chinese college students by translating abstract competencies into observable daily behaviors (e.g., academic-database retrieval, misinformation identification, online collaboration, privacy protection, digital content creation).

Before the formal survey, the initial 12-item digital-literacy pool was reviewed by four experts: two scholars in digital education, one in educational/psychological measurement, and one in student development. Each expert rated every item on a four-point relevance scale (1 = not relevant to 4 = highly relevant). In the first round, I-CVI ranged from 0.75 to 1.00 across the 12 items. Two items (DL3 on keyword retrieval and DL8 on online risk prevention) received I-CVI = 0.75 (three of four experts rated them relevant); their wording was revised and re-rated. Final I-CVI across the 12 items ranged from 0.75 to 1.00, with four items retaining I-CVI = 0.75 after revision (the two revised items DL3 and DL8, together with two further items that were retained because their content was judged relevant by three of four experts). The scale-level CVI, computed as the average of item-level CVI values (S-CVI/Ave), was 0.92. We report S-CVI/Ave rather than S-CVI/UA because universal agreement across all 12 items was not achieved.

We acknowledge that I-CVI values of 0.75 are below the commonly recommended threshold for a four-expert panel and therefore represent a limitation in the content-validity evidence. Specifically, DL3 captures individuals' ability to locate relevant digital information efficiently, whereas DL8 reflects protective competence in digital environments, both of which correspond to the functional and safety dimensions of digital literacy. These items were provisionally retained because their content represented theoretically relevant aspects of the intended construct that would be underrepresented if they were deleted. Subsequent exploratory factor analysis and internal-consistency assessment were used to examine the psychometric performance of the retained items, but these analyses were not treated as substitutes for expert content-validity evaluation. Further expert assessment and independent validation in larger samples are warranted.

Following expert review and item revision, a pilot test was conducted with 60 undergraduates recruited from three universities that were not part of the formal survey sample. The pilot evaluated item clarity, response distribution, completion time, and internal consistency. Respondents completed the pilot in a mean of 8.4 min (SD = 2.1), and Cronbach's alpha for the 12-item digital-literacy composite was 0.79. Three items (DL1, DL5, DL9) were flagged by at least two pilot respondents as slightly ambiguous; their wording was minimally revised before formal data collection. Because the items were originally developed in Chinese for Chinese college students rather than translated from a foreign-language scale, no forward-back translation procedure was applicable; the English items in this paper are reporting translations.

### 2.2 Positive psychological functioning: conceptualization and measurement

In this study, the outcome is termed “positive psychological functioning” to distinguish the study-specific composite from established clinical mental-health or psychological-wellbeing measures. Positive psychological functioning is conceptualized as a state of positive psychological functioning and adaptive adjustment rather than as the absence of clinical symptoms.

Positive psychological functioning is conceptually distinguished from adjacent constructs. It differs from symptom-oriented measures such as the CES-D, which primarily assess psychological distress, and from brief hedonic wellbeing measures such as the WHO-5. It also differs from Ryff's (1989) psychological wellbeing model, which emphasizes eudaimonic outcomes such as purpose in life, personal growth, and environmental mastery in the general adult population (Ryff, 1989). Positive psychological functioning here is closer to the positive-psychology and college-adaptive-functioning tradition, emphasizing current functioning resources and adaptive capacities rather than stable personality traits or long-term eudaimonic wellbeing (Costa and McCrae, 1992). The six dimensions, namely emotional functioning, volitional functioning, self-awareness, personality integration, interpersonal functioning, and adaptive adjustment, are intended to capture context-sensitive psychological resources relevant to college students' daily academic and social functioning.

Established instruments such as the Center for Epidemiologic Studies Depression Scale (CES-D), the World Health Organization WellBeing Index (WHO-5), or Ryff's Psychological WellBeing Scales were considered but not adopted. CES-D and WHO-5 primarily capture psychological distress or hedonic wellbeing, whereas this study focuses on a broader set of college-adaptive outcomes including academic self-regulation, interpersonal functioning, and behavioral adjustment. Ryff's multidimensional wellbeing scales cover conceptually adjacent domains but were developed for general adult populations and include items less suited to the college-student context in Henan Province. A study-specific composite was therefore constructed to match the target population and the adaptive-functioning framework underlying the study, while the measurement limitations of this choice are acknowledged in the Limitations section.

(1) Emotional health refers to the capacity to maintain emotional stability, sustain positive attitudes, and deploy adaptive coping strategies under stress. It reflects both the absence of persistent negative affect and the presence of optimistic cognitive patterns and emotion-regulation skills.

(2) Volitional functioning captures goal-directedness, resilience in the face of setbacks, and sustained execution of study plans. It reflects the degree to which students maintain clear plans, recover from failures, and persist in goal pursuit.

(3) Self-awareness concerns the accuracy of self-evaluation, openness to feedback, and the ability to select roles and tasks that match one's capabilities. It reflects objective self-knowledge and appropriate role positioning in social and academic contexts.

(4) Personality integration refers to the consistency of values and behavioral patterns across situations, and the coordination among cognition, emotion, and behavior. It reflects a stable and integrated personality structure rather than fragmentation or situational inconsistency.

(5) Interpersonal relationships assess the quality of social interaction, empathic sensitivity, and collaborative efficacy. It captures both conflict resolution skills and the ability to provide and receive social support in peer relationships.

(6) Adaptability reflects the speed and quality of adjustment to new environments, new learning modes, and cultural differences. It captures students' capacity to adapt to changing living, learning, and social circumstances.

Based on this conceptualization, a study-specific 18-item positive-psychological-functioning composite was constructed, with three items per dimension (Table 2). All items were rated on a five-point Likert scale ranging from 1 (“strongly disagree”) to 5 (“strongly agree”). Negatively worded items were reverse-coded so that higher values consistently indicated better psychological functioning. Raw dimension scores were computed as the sum of the three items assigned to each dimension. The overall positive psychological functioning score and each dimension score were then linearly transformed to a 0–100 scale. For the overall scale (18 items, each 1–5), the minimum possible sum is 18 and the maximum is 90; for each three-item subscale, the minimum is 3 and the maximum is 15. This linear transformation preserves the relative ordering of respondents while placing all scores on a common 0–100 metric for ease of interpretation. The same transformation procedure was applied to the digital-literacy scale for comparability.

**Table 2:** Comprehensive evaluation index system of college students' positive psychological functioning.

Primary indicator	Secondary indicator	Tertiary indicator	Specific manifestation
Emotional health	Emotional stability	Stress-related emotional disturbance	Has insomnia or decreased appetite due to academic stress in the past week
	Positive attitude	Optimistic cognitive ability	When encountering setbacks, I tend to focus on positive solutions
	Emotional regulation ability	Stress coping strategies	When emotionally agitated, I regulate through exercise/confiding, etc.
Volitional functioning	Goal orientation	Career planning clarity	I have a clear future career development plan
	Resilience to setbacks	Resilience in the face of difficulties	When encountering project failure, I analyze the reasons and adjust strategies
	Action sustainability	Goal execution intensity	I can persist in completing my study plan every day
Self-awareness	Accuracy of self-awareness	Ability to identify strengths and weaknesses	I can clearly list three personal strengths and two areas for improvement
	Objectivity of evaluation	Acceptance of others' feedback	When classmates point out my shortcomings, I analyze rationally
	Role appropriateness	Task selection matching	In club activities, I take the initiative to undertake tasks that match my abilities
Personality integration	Consistency of values	Stability of moral behavior	My integrity standards remain consistent in different situations (such as exams/social interactions)
	Stability of behavioral patterns	Consistency in situational responses	My reaction patterns are predictable when facing unexpected situations
	Coordination of psychological elements	Coordination of cognition-emotion-behavior	My thoughts, feelings, and actions are usually unified
Interpersonal relationships	Interaction quality	Conflict resolution ability	When conflicts arise with roommates, I can reach a consensus through communication
	Empathy ability	Sensitivity to emotions	I can promptly detect changes in classmates' emotions and provide support
	Cooperative efficacy	Team contribution	In group assignments, I take the initiative to undertake tasks and coordinate team members
Adaptability	Environmental adaptation	Speed of adaptation to new situations	When moving to a new living environment, I can establish a new routine within a week
	Learning adaptation	Transition ability in learning methods	I can quickly master new teaching models such as MOOCs and flipped classrooms
	Social adaptation	Cultural difference tolerance	In the face of different living habits of students from different regions, I can maintain respect

The expert review supported the intended mapping of the six-domain structure onto the study-specific student-adaptive-functioning construct rather than a clinical mental-health measure; given the small expert panel and several item-level CVI values of 0.75, this evidence is treated as preliminary rather than definitive.

The same 60-respondent pilot also evaluated the 18-item Positive psychological-functioning composite, for which Cronbach's alpha was 0.81. Two positive psychological-functioning items (PPF3, PPF11) were slightly revised for clarity based on pilot feedback.

### 2.3 The association between digital literacy and college students' positive psychological functioning

This study develops an integrated digital capability-resource-regulation framework. Digital literacy is treated as a broad enabling capability rather than a single technical skill (Zhao et al., 2024). It may be related to positive psychological functioning through three proximal processes: cognitive regulation of digital information, access to relational resources in online environments, and self-regulatory digital behavior. Information processing theory explains how students select, evaluate, and organize digital information; social support theory explains how digital competence can facilitate supportive interaction and a sense of connection; and cognitive-behavioral and self-determination perspectives jointly explain how digital competence may strengthen perceived control, task competence, and adaptive behavior. These theories are therefore used as complementary components of one framework rather than as independent explanations.

The five dimensions of digital literacy provide different inputs into these processes. Digital tool use proficiency supports efficient task completion and perceived competence. Information acquisition and processing primarily contributes to cognitive regulation, although intensive information exposure may also increase cognitive load. Digital social interaction is most directly linked to relational resources and social support. Digital innovation may strengthen competence and creative expression, but it may also generate performance pressure and demands for continuous skill updating. Digital security and ethical awareness may reduce exposure to online risks, although its relevance to positive psychological functioning may be indirect or conditional on actual risk exposure. This mapping allows the overall association to coexist with different, and potentially opposite, dimension-specific associations.

At the overall level, students with stronger digital literacy may be better able to use digital resources purposefully, solve learning-related problems, manage digital risks, and maintain constructive online relationships. These capacities may reduce avoidable stress and strengthen perceived control. Accordingly, the study proposes the following hypothesis:

Hypothesis H1: Overall digital literacy is positively associated with college students' positive psychological functioning.

The first decomposition component concerns digital information regulation behavior. Information processing theory posits that the human cognitive process is similar to a computer's information processing system, involving the input, encoding, storage, retrieval, and output of information (Early, 1976). In digital environments, digital literacy may shape students' ability to process information effectively. Students with higher digital literacy may be better able to filter, analyze, and integrate online information, which may help limit cognitive stress associated with information overload (Zhao et al., 2024). They may use critical thinking to evaluate information and discern its authenticity and value, which may reduce exposure to psychologically burdensome or misleading information. When confronted with a plethora of health information online, Students with higher digital literacy may be better positioned to evaluate the reliability of online health information and identify more credible guidance, which may be associated with more adaptive psychological functioning. In contrast, students with lower digital literacy may be misled by false information, leading to negative emotions such as anxiety and panic.

Exploratory Expectation E1: Digital information regulation behavior may statistically account for part of the cross-sectional association between digital literacy and positive psychological functioning.

The second decomposition component proposes that digital literacy may be associated with positive psychological functioning (psychological functioning) partly through online social boundary practices. As digital environments become increasingly central to academic and social life, students with higher digital literacy are better equipped to navigate complex interpersonal dynamics across multiple platforms (Fasihi Harandi et al., 2017). Specifically, digital literacy may support three interrelated competencies: effective collaborative communication in online learning and group work contexts, strategic self-presentation that aligns with normative expectations across different social media and professional platforms, and proactive boundary management that protects personal privacy and regulates the intensity of digital social contact.

These social boundary practice competencies, in turn, shape key psychosocial resources relevant to psychological functioning. By facilitating clearer communication and more coordinated collaboration, digitally literate students are more likely to secure instrumental and emotional support from peers, instructors, and broader online communities. Furthermore, deliberate boundary-setting and self-presentation strategies may help reduce the likelihood of social comparison stress, identity confusion, and relational ambiguity that often accompany unregulated online engagement. From a social support perspective, the quality and accessibility of social resources function as a protective buffer against psychological distress, while reduced relational uncertainty contributes to greater emotional stability and lower anxiety (Antonucci, 1985). Digital social interaction represents the potential or ability, whereas online social boundary practice captures the downstream application and regulatory practice of that ability in concrete social contexts.

Exploratory Expectation E2: Online social boundary practice may statistically account for part of the cross-sectional association between digital literacy and positive psychological functioning.

The third decomposition component centers on structured digital use practice and self-regulation as an underlying exploratory decomposition link connecting digital literacy to positive psychological functioning. Digital literacy encompasses not only declarative knowledge but also procedural skills that translate into organized, goal-directed digital behavior. Students with stronger digital literacy demonstrate greater operational proficiency in using digital tools, more systematic approaches to organizing and managing digital information and materials, and more intentional and creative engagement with digital content rather than passive consumption (Jameel et al., 2025).

These enacted behavioral patterns contribute to psychological wellbeing through two complementary theoretical lenses. First, from a cognitive-behavioral perspective, mastery experiences and perceived behavioral control are foundational to adaptive coping (Thoma et al., 2015). When students can efficiently navigate digital environments, organize their digital lives, and produce purposeful content, they develop a sense of competence and agency that generalizes to broader life domains. Second, self-determination theory posits that satisfaction of the basic psychological need for competence is a critical driver of intrinsic motivation and psychological wellness (Deci and Ryan, 2012). Purposeful digital creation and structured digital management provide repeated opportunities for competence satisfaction, which in turn support autonomous motivation, goal-directed behavior, and overall psychological adjustment.

Exploratory Expectation E3: Structured digital use practice may statistically account for part of the cross-sectional association between digital literacy and positive psychological functioning.

Because the decomposition constructs are conceptually related to dimensions of digital literacy, the analysis is exploratory and is interpreted as association decomposition rather than causal mediation. No causal claim is made.

## 3 Research design

### 3.1 Model design

The empirical analysis first estimates the association between digital literacy and positive psychological functioning using the following cross-sectional model (Equation 1):


$$\begin{array}{l} PPF_{i}=\alpha +\beta DL_{i}+\gamma X_{i}+\varepsilon_{i} \end{array}$$ (1)

where *PPF* denotes the positive-psychological-functioning score of student *i*,

Dl denotes the overall digital-literacy score, *X* is a vector of individual, educational, and social control variables, and ε is the error term. The coefficient β is interpreted as a conditional association rather than a causal effect.

For regression coefficients, small-cluster inference was assessed using the wild-cluster bootstrap with Webb weights and 1,000 replications. For the product-of-coefficient indirect components in Table 3, uncertainty was assessed separately using 2,000 cluster-resampling bootstrap replications at the university level. Bootstrap results for the baseline models are reported in Appendix Table B; wild-cluster bootstrap *p*-values for all secondary models are reported in Appendix Table E.

**Table 3:** Pathway decomposition.

Pathway construct	Information regulation	Positive psychological functioning	Social boundary	Positive psychological functioning	Structured use	Positive psychological functioning
	(1)	(2)	(3)	(4)	(5)	(6)
Digital literacy	0.125^***^	0.028^***^	0.191^***^	0.063^**^	0.201^***^	0.058^*^
	(4.25)	(3.25)	(5.84)	(2.24)	(5.31)	(1.96)
Pathway construct		1.201^***^		0.602^***^		0.597^***^
		(4.02)		(4.65)		(5.21)
95% CI	[0.082, 0.231]		[0.064, 0.179]		[0.069, 0.187]	
Indirect effect	0.150		0.115		0.120	
Wild-boot *p* for indirect	< 0.001		< 0.001		< 0.001	
Control variables	YES	YES	YES	YES	YES	YES
*N*	893	893	893	893	893	893
*R* ^2^	0.284	0.052	0.351	0.048	0.312	0.045

Values in parentheses are *t* statistics based on university-clustered standard errors. Wild-cluster bootstrap *p*-values for the indirect components are based on 1,000 Webb-weight replications, whereas bias-corrected 95% confidence intervals are based separately on 2,000 university-level cluster-resampling bootstrap replications. All models include the full set of control variables. ^***^, ^**^, and ^*^ denote *p* < 0.01, *p* < 0.05, and *p* < 0.10, respectively; Because the data are cross-sectional and the constructs partially overlap, the results are interpreted as pathway decomposition rather than causal mediation. Because the digital-literacy index, pathway constructs, and positive-psychological-functioning outcome are measured on different scales, the indirect components are scale-dependent product-of-coefficient estimates and should not be compared as standardized effect sizes or interpreted as proportions of a total effect.

For each proposed decomposition component *k*, the total association was decomposed into a direct component and an indirect component using Equations (2) and (3):


$$\begin{array}{c} M_{ki}=\alpha_{k}+\alpha_{k}DL_{i}+\gamma_{k}X_{i}+\varepsilon_{ki} \end{array}$$ (2)

$$\begin{array}{c} PPF_{i}=\alpha +c_{k}\beta DL_{i}+b_{k}M_{k}+\gamma X_{i}+\varepsilon_{i} \end{array}$$ (3)

where *M*_*ki*_ denotes the *k*_*ik*_ pathway construct. The indirect component was calculated as *a*_*k*_*b*_*k*_, and its statistical uncertainty was evaluated using 2,000 university-level cluster-resampling bootstrap replications, with universities resampled as clusters. Bias-corrected 95% confidence intervals were obtained from 2,000 university-level cluster-resampling bootstrap replications. All decomposition results are interpreted as exploratory associational decomposition, not causal mediation.

The instrumental-variable sensitivity analysis using city-level internet penetration is moved to Appendix Table C. Due to untestable exclusion restriction and weak-identification risk, IV results serve purely as supplementary sensitivity check and cannot resolve endogeneity or support causal inference.

### 3.2 Data variables

The data were collected in 2024 through a cross-sectional survey of college students in Henan Province, China. Recruitment was conducted through Questionnaire Star, an online survey platform, with IP geofiltering restricted to Henan Province. The 18 universities were not selected from a probability-based institutional sampling frame before data collection. After quality screening, the analytical sample comprised 893 respondents affiliated with 18 universities in Henan Province. University affiliation was determined from respondents' self-reported institutional information. The institutions were not randomly sampled, and the sample should therefore be interpreted as a nonprobability sample rather than a representative sample of all university students in the province. Within-participant eligibility considered grade, gender, and major through self-report. Before participation, respondents received an explanation of the study purpose, voluntary participation, confidentiality, and completion requirements. Of the 1,200 students invited to participate, 1,053 submitted completed responses by the survey deadline. Responses were excluded according to prespecified quality-control criteria: (a) more than 10% item-level missingness (*n* = 46); (b) completion time below 5 min, which fell below one-third of the median completion time (15.6 min) in the returned sample (*n* = 39); (c) logically inconsistent responses on predefined paired items (*n* = 31). Two types of direct contradictions were checked: responses indicating no use of a smartphone for learning while simultaneously reporting frequent use of online collaborative tools, and responses reporting no prior use of academic databases while simultaneously reporting frequent literature retrieval. A response was excluded if it exhibited either direct contradiction, because such discrepancies cannot plausibly reflect consistent self-reported behavior. Other discrepancies between self-reported capabilities, experiences, and behaviors were not treated as grounds for exclusion, because they may reflect meaningful variation rather than careless responding; and (d) straight-lining, defined as 15 or more consecutive identical responses on Likert items combined with a within-person standard deviation below 0.25 across all rating items (*n* = 44). Where multiple criteria were met, cases were assigned to a prespecified hierarchical category ordered from (a) to (d) to avoid double counting; in total, of the 1,053 returned questionnaires, 160 were excluded during data-quality screening, resulting in 893 valid observations for analysis. The proportion of completed responses relative to distributed invitations was 87.75%, and the valid-response rate was 74.4%.

The 18 participating universities comprised 6 comprehensive universities, 5 normal (teacher-training) universities, 3 engineering-oriented universities, 1 financial and economics universities, and 3 medical or agricultural universities. Valid responses per institution ranged from 32 to 78, with a median of 49. Within the final sample, 58.3% identified as female, 41.7% as male; respondents were distributed across first-year (23.8%), second-year (28.6%), third-year (27.4%), and fourth-year (20.2%) cohorts; and 61.4% majored in humanities or social sciences, 28.9% in science or engineering, and 9.7% in medical or agricultural fields.

No formal a-priori power analysis was conducted at the time of survey design. Recruitment sought to maximize eligible responses during the prespecified survey period (February–May 2024), and the final sample of 893 was determined by the available response volume rather than by a pre-specified effect size. We acknowledge this as a design limitation. Future research using probability-based sampling and pre-specified power calculations is needed to confirm the present findings.

To strengthen construct validation, the full sample was randomly divided into a calibration subsample (*n* = 446) for exploratory factor analysis and a validation subsample (*n* = 447) for confirmatory factor analysis. The split-sample results provide mixed rather than unequivocal support for the proposed measurement structure. In the calibration subsample, the exploratory solution broadly differentiated the digital-literacy domains and several positive-psychological-functioning domains, but personality integration, interpersonal relationships, and adaptability were not cleanly separated into distinct factors. Selected primary and salient cross-loadings from the split-sample EFA, together with standardized CFA loadings and model-fit indices, are reported in Appendix Table A. In the validation subsample, confirmatory factor analysis of the theoretically specified measurement model showed acceptable overall fit (χ^2^/df = 2.31, CFI = 0.91, TLI = 0.90, RMSEA = 0.054, SRMR = 0.061). Convergent validity provided partial support: CR values were at or above 0.70 for most scales, but average variance extracted (AVE) values ranged from 0.39 to 0.58 across the assessed constructs, with several constructs falling below the conventional 0.50 threshold. Discriminant validity was also only partially supported, particularly for the decomposition constructs. Accordingly, the overall composite indices are used as the principal measures, whereas dimension-specific and decomposition analyses are interpreted as exploratory.

Internal consistency was evaluated using Cronbach's alpha, composite reliability (CR), and Spearman-Brown coefficients for the two-item subscales. The Cronbach's alpha coefficients were 0.824 for the overall 12-item digital-literacy scale, and 0.731, 0.758, 0.742, 0.705, and 0.719 for its five dimensions. For the three two-item subscales, namely digital tool use proficiency, digital innovation, and digital security and ethical awareness, the Spearman-Brown coefficients were 0.738, 0.712, and 0.710, respectively. For the positive-psychological-functioning scale, the overall alpha was 0.862, with subscale alphas of 0.784, 0.756, 0.723, 0.741, 0.775, and 0.738. The three pathway constructs had alphas of 0.687, 0.712, and 0.694. The corresponding CR values ranged from 0.692 to 0.857. Most scales met or approached the conventional reliability threshold of 0.70. Two of the three-item pathway constructs had alpha coefficients below 0.70 (0.687 and 0.694), indicating relatively limited internal consistency. Results involving these constructs should therefore be interpreted cautiously.

Construct validity was examined using exploratory factor analysis with principal axis factoring and promax rotation, because the proposed constructs were theoretically expected to be correlated. The KMO statistic was 0.847, and Bartlett's test of sphericity was significant, χ^2^(741) = 8,926.3, *p* < 0.001, indicating that the data were suitable for factor analysis. Factors were retained based on the eigenvalue-greater-than-one rule in conjunction with the scree plot inspection. The exploratory factor solution broadly differentiated the five digital-literacy domains and several positive-psychological-functioning domains, but it did not reproduce the full prespecified measurement structure cleanly. In particular, personality integration, interpersonal relationships, and adaptability showed substantial empirical overlap in the calibration subsample. Pattern coefficients on the dominant factors ranged from 0.523 to 0.841, and no retained item showed a secondary pattern coefficient above 0.40. These EFA results therefore provide partial, rather than complete, support for the proposed multidimensional structure. The retained solution explained 62.4% of the total variance. Harman's single-factor test was conducted to assess common method bias based on all measurement items. The first unrotated factor explained 26.1% of the total variance, suggesting that no single factor dominated the covariance structure. However, Harman's single-factor test is only a descriptive diagnostic and cannot rule out common-method bias. Common-method variance therefore remains a limitation of the self-report, cross-sectional design.

Convergent validity provided partial support: CR values were at or above 0.70 for most scales, but average variance extracted (AVE) values ranged from 0.39 to 0.58, with several constructs falling below the conventional 0.50 threshold. Discriminant validity was only partially supported. For two of the three pathway constructs, the square root of AVE slightly exceeded the correlation with the overall digital-literacy index, whereas the remaining construct did not fully satisfy the Fornell-Larcker criterion. The pathway constructs were therefore treated as conceptually and empirically overlapping with the overall digital-literacy index. Multicollinearity was examined using variance inflation factors (VIF) across the main regression models; VIF values ranged from 1.12 to 2.38, with a maximum of 2.38 for the overall digital-literacy predictor, well below the conventional 10.0 cutoff and indicating no serious multicollinearity concern. Sensitivity analyses using factor-score and entropy-weighted digital-literacy indices were conducted to assess the robustness of the main digital-literacy-positive psychological functioning association.

(1) Dependent Variable

The dependent variable is the composite positive psychological functioning score. The scale contains 18 items across six dimensions: emotional health, volitional functioning, self-awareness, personality integration, interpersonal relationships, and adaptability. Responses were coded from 1 to 5. Negatively worded items were reverse-coded so that higher values consistently represented better positive psychological functioning. The total score was calculated by summing the item scores and transforming the result to a 0–100 scale. Subscale scores were calculated using the same coding direction.

(2) Core Independent Variable

The core independent variable is the composite digital-literacy score. The scale contains 12 items across five dimensions: digital tool use proficiency, information acquisition and processing, digital social interaction, digital innovation, and digital security and ethical awareness. Each item was rated from 1 to 5, and the overall score was calculated by summing all items and transforming the result to a 0–100 scale. Higher values indicate stronger digital literacy. Dimension scores were constructed from the items assigned to each dimension and were analyzed separately in the dimension-specific models.

(3) Pathway Variables

Three decomposition constructs were operationalized to represent information regulation, online social-boundary management, and behavioral self-regulation in digital environments. An important caveat is that these constructs show moderate-to-high bivariate correlations with overall digital literacy (0.58–0.65), and discriminant validity is only partially supported; they are therefore not fully empirically distinct from the digital-literacy dimensions.

Digital information regulation behavior captures students' applied judgment of digital information in daily use, comprising three indicators. Initiative filtering: “I actively filter out low-value or misleading information during daily online browsing”. Information load control: “I consciously control the amount of information I receive to avoid cognitive overload”. Source verification: “I verify the source of information before accepting or applying it in my studies”.

Online social boundary practice reflects strategic regulation of social behavior and relational boundaries across digital platforms. The construct comprises three items. Communication boundary setting: “I set clear communication boundaries when collaborating with others via digital tools”. Cross-platform self-management: “I manage my self-presentation differently across social and academic digital platforms”. Privacy protection practice: “I regularly adjust privacy settings to protect my personal information online”.

Structured digital use practice captures concrete operational practices and self-regulatory strategies in digital environments. The construct comprises three items. Digital task planning: “I use digital tools to systematically organize my daily study tasks and schedules”. Digital material archiving: “I regularly sort and archive digital learning materials to keep them organized”. Usage time management: “I arrange my daily digital usage time in a planned and purposeful manner.”

Given the conceptual overlap between each pathway construct and the corresponding digital-literacy dimension, several diagnostic checks were conducted. The bivariate correlations between the overall digital-literacy index and the three pathway constructs were 0.62 for digital information regulation behavior, 0.58 for online social boundary practice, and 0.65 for structured digital use practice. VIF values in the pathway models ranged from 1.41 to 2.38, indicating that multicollinearity did not severely destabilize the estimates. However, discriminant validity was only partially supported. The results are interpreted as exploratory decomposition evidence rather than proof of distinct causal mechanisms.

(4) Control Variables

According to the health capital theory and relevant literature research experience, this paper sets two types of control variables at the individual and social levels (Grossman, 1972; Li and Yang, 2024). The control variables include age, gender, household registration, exercise frequency, monthly living expenses (kept as continuous variable for moderation tests), major, university type, and social trust. Gender is coded 0 for female and 1 for male. Household registration is coded 0 for rural and 1 for urban. Major was coded 0 for science, engineering, and medicine and 1 for humanities and social sciences. University type was coded 0 for public universities and 1 for private universities. Monthly living expenses were measured in thousands of Chinese yuan; therefore, a one-unit increase represents an additional CNY 1,000 per month. The coding and reference categories are reported in the descriptive-statistics table.

### 3.3 Descriptive statistical analysis

Table 4 summarizes the distributions of the study variables. On the 0–100 scale, the mean positive-psychological-functioning score was 60.3 (SD = 11.6; range = 24–91), and the mean digital-literacy score was 78.6 (SD = 8.7; range = 47–95). Because neither scale has a validated diagnostic or categorical cutoff, the scores are interpreted as relative levels within the present sample rather than as low, medium, or high categories.

**Table 4:** Descriptive statistics.

Variable	Definition	Mean	SD	Min	Max
Positive psychological functioning	Composite score, 18 items across six dimensions, linearly transformed to a 0–100 scale	60.32	11.6	24	91
Digital literacy	Composite score, 12 items across five dimensions, linearly transformed to a 0–100 scale	78.63	8.73	47	95
Digital information regulation behavior	Sum of three items (raw score range: 3–15)	12.51	1.72	7	15
Online social boundary practice	Sum of three items (raw score range: 3–15)	10.93	1.54	8	15
Structured digital use practice	Sum of three items; raw score range: 3–15	8.94	1.75	6	14
Age	Actual age in the year	19.42	1.37	17	23
Gender	Female 0, Male 1	0.47	0.50	0	1
Household registration	Rural household registration 0, Urban household registration 1	0.70	0.46	0	1
Exercise	Frequency of physical exercise per week	7.15	2.98	0	14
Major	science and engineering, medicine = 0; humanities and social sciences = 1	0.46	0.50	0	1
University type	Public university = 0, private university = 1	0.39	0.49	0	1
Living expenses	Monthly living expenses, measured in thousands of Chinese yuan	1.25	0.41	0.8	3
Social trust	Sum of trust levels toward classmates and strangers	6.12	0.68	5	8

The positive psychological functioning score was transformed as (*Raw* score−18)/(90−18) × 100, and the 12-item digital-literacy score was transformed as (*Raw* score −12)/(60−12) × 100. These transformations used the theoretical minimum and maximum scores rather than the observed sample range. The three pathway variables are reported as raw sum scores ranging from 3 to 15. The analytical sample included 893 respondents.

## 4 The association between digital literacy and college students' positive psychological functioning

Descriptive statistics were first calculated for all variables, including the mean, standard deviation, minimum, and maximum. Pearson correlations and variance inflation factors were used to assess bivariate relationships and multicollinearity. The main association was estimated using ordinary least squares regression with clustered standard errors (clustered at the university level). Robustness analyses used factor-score and entropy-weighted indices. Heterogeneity was examined using full-sample interaction models for household registration, gender, living expenses, major, and city type, together with quadratic and spline specifications for potential non-linearity in digital literacy. Because multiple subgroup tests increase the risk of false-positive findings, these results are interpreted as exploratory; Benjamini-Hochberg false-discovery-rate adjustment is applied to the three test families (dimensions, sub-dimensions, interactions) and reported in Appendix Table F.

### 4.1 Benchmark regression results

Based on model (1), the regression results obtained using the ordinary least squares method are shown in Table 5.

**Table 5:** The association between digital literacy and positive psychological functioning.

Variable	Positive psychological functioning			
	Unadjusted OLS	Adjusted OLS	Factor-score index	Entropy-weighted index
	(1)	(2)	(3)	(4)
Digital literacy	0.216^***^	0.178^***^	0.162^***^	0.169^***^
	(14.52)	(5.14)	(4.91)	(4.97)
Age		0.157	0.132	0.147
		(0.49)	(0.57)	(0.95)
Gender		0.168^*^	0.152	0.157
		(1.91)	(1.63)	(1.42)
Household registration		1.58^**^	1.82^**^	1.41^**^
		(2.23)	(2.18)	(2.39)
Exercise		1.325^***^	1.014^***^	1.154^***^
		(7.19)	(5.98)	(4.95)
Humanities and social sciences		0.912	0.715	0.547
		(0.97)	(1.12)	(1.31)
Private university		0.325	0.319	0.351
		(1.25)	(1.03)	(1.54)
Living expenses		0.092^**^	0.107^**^	0.131^***^
		(2.49)	(2.36)	(2.95)
Social trust		0.057^***^	0.024^***^	0.054^***^
		(2.98)	(4.12)	(5.36)
*N*	893	893	893	893
*R* ^2^	0.026	0.031	0.028	0.051

Values in parentheses are *t* statistics. Standard errors in columns (1)–(4) are clustered at the university level (18 clusters). The 18 universities were located in 17 cities. ^**^p < 0.05, ^***^p < 0.01.

Two qualifications are essential. First, the explanatory power of the full adjusted model is modest (*R*^2^ = 0.031), indicating that the included predictors collectively explain only a small proportion of the observed variation in positive psychological functioning. Second, because the data are cross-sectional, β = 0.178 should be interpreted as a conditional association rather than a causal effect. The result is therefore consistent with Hypothesis H1 at the associational level only.

Columns (3) and (4) re-estimate the model using alternative digital-literacy indices constructed through factor scoring and entropy weighting. Both specifications yielded positive and statistically significant coefficients, indicating that the direction and statistical inference of the association were robust to alternative index construction.

As a further small-sample robustness check, we re-estimated statistical significance for the three baseline models (OLS, factor-score index, entropy-weighted index) using the wild-cluster bootstrap with Webb six-point weights and 1,000 replications clustered at the university level. As reported in Appendix Table B, all three coefficients remain significant at the 1% level under this correction.

The instrumental-variable analysis using city-level internet penetration as the instrument is moved to Appendix Table C and is presented strictly as a supplementary sensitivity check. The first-stage Kleibergen-Paap rk Wald F statistic was 12.89. Although this value exceeds the conventional rule-of-thumb threshold of 10, it does not eliminate weak-identification concerns, particularly given the limited number of clusters. The IV estimate is reported solely as a supplementary sensitivity analysis and is not used to strengthen causal interpretation of the OLS association.

### 4.2 Dimension-specific associations between digital literacy and positive psychological functioning

Each digital-literacy dimension was entered into a separate model to avoid multicollinearity among closely related dimensions. Accordingly, each coefficient represents the association between one dimension and positive psychological functioning after adjustment for the control variables; because the five dimensions are estimated in separate models, the coefficients should not be interpreted as mutually adjusted or independent net effects.

In column (1), digital tool use proficiency had a positive coefficient, but the association was not statistically significant. Therefore, the result should not be interpreted as evidence of a statistically detectable association between digital tool use proficiency and positive psychological functioning in the present sample.

In column (2), information acquisition and processing was negatively associated with positive psychological functioning (psychological functioning). After wild-cluster bootstrap inference and FDR adjustment, this result remains statistically significant. This association may be explained by greater exposure to conflicting information and higher cognitive load among students who engage in more frequent information searching and integration. Against the background of massive and uneven-quality online information, frequent information acquisition and processing may increase the cognitive burden of information screening and value judgment, which corresponds to higher psychological tension (Sheng et al., 2023). Reverse causality is also possible: students experiencing more psychological distress may conduct more online information seeking in an effort to reduce uncertainty. The cross-sectional design cannot distinguish these explanations.

In column (3), digital social interaction was positively associated with positive psychological functioning. This result survives wild-bootstrap and FDR adjustment. This result aligns with the perspective that digital social competence may help students maintain and expand interpersonal connections, and obtain more emotional and instrumental support through online channels, which is related to better psychological status (Zhang, 2023). However, this study cannot confirm the causal direction among digital social interaction, social support acquisition and positive psychological functioning.

In column (4), digital innovation carried a negative coefficient under conventional clustered inference, but the association was not statistically significant under wild-cluster bootstrap (*p* = 0.116) or BH-FDR adjusted (*q* = 0.193) inference. It is therefore treated as a non-robust exploratory pattern rather than as evidence of a negative association. A plausible explanation is that digital innovation practices such as digital content creation and cross-domain technical application are often accompanied by continuous skill updating requirements and self-evaluation pressure, which may bring additional time and mental burden to students, thus showing a negative correlation with positive psychological functioning (Seiferth et al., 2023). It is also possible that students facing higher academic or career pressure are more motivated to engage in digital innovation. The cross-sectional data cannot clarify the temporal order of the two.

In column (5), digital security and ethical awareness was not statistically associated with positive psychological functioning. One possible reason is that security and ethical awareness is a relatively distal competence, and its protective effect for psychological functioning may only manifest when students actually encounter online risks such as privacy leakage, fraud or cyberbullying. In the absence of direct risk exposure, awareness level alone may not be significantly reflected in daily psychological functioning (Ramos-Soler et al., 2018).

In addition, the potential disconnect between awareness and actual behavioral practice may further weaken the association between this dimension and positive psychological functioning. After both small-cluster correction and multiple-testing correction, tool use, security, and innovation no longer meet the 5% threshold, so we describe them as exploratory patterns rather than robust findings.

### 4.3 Associations between digital literacy and positive psychological functioning dimensions

Just as averaging five digital-literacy dimensions can hide heterogeneity, the single 18-item positive-psychological-functioning composite score also aggregates six distinct outcomes. We therefore re-run the baseline model six times, once for each sub-dimension (emotional health, volitional functioning, self-awareness, personality integration, interpersonal relationships, adaptability), to see which specific aspects of psychological functioning track digital literacy most closely. Because these regressions also rely on university-clustered standard errors with only 18 clusters, we apply the same two corrections as in Table 6: wild-cluster bootstrap *p*-values (correcting small-cluster bias) and Benjamini-Hochberg FDR-adjusted *p*-values (correcting for six parallel tests). The specific results are shown in Table 6.

**Table 6:** Dimension-specific associations of digital literacy with positive psychological functioning.

Variable	Digital tool use proficiency	Information acquisition and processing	Digital social interaction	Digital innovation	Digital security and ethical awareness
	(1)	(2)	(3)	(4)	(5)
Dimension score	0.095	−0.157^***^	0.075^***^	−0.057^*^	0.071
	(1.64)	(−4.95)	(7.13)	(−1.79)	(1.05)
Wild-bootstrap *p*	0.148	0.003	0.001	0.116	0.347
BH-FDR *q*	0.193	0.008	0.005	0.193	0.347
Control variables	YES	YES	YES	YES	YES
*N*	893	893	893	893	893
*R* ^2^	0.028	0.035	0.041	0.027	0.026

Each dimension entered in a separate model to avoid within-construct multicollinearity. *t*-statistics in parentheses. Wild-cluster bootstrap *p*-values (Webb six-point, 1,000 reps) and Benjamini-Hochberg FDR-adjusted *p*-values across the 5 dimension tests are shown. ^***^, ^**^, and ^*^ denote *p* < 0.01, *p* < 0.05, and *p* < 0.10, respectively.

In column (1), digital literacy was positively associated with emotional health. One possible interpretation is that students with higher digital literacy can access wellbeing information and emotional support resources more conveniently through digital channels, which helps them maintain emotional stability and adopt adaptive coping styles when facing stress (Wilhelm et al., 2020). This correlational finding does not confirm a specific intervention pathway, and the underlying process requires further examination using longitudinal data.

In column (2), digital literacy was positively associated with volitional functioning. This result may be related to the auxiliary role of digital literacy in task management and goal planning: proficient use of digital tools may help students decompose learning tasks and arrange progress systematically, thus maintaining better execution and resilience in the face of setbacks (Tarifa-Rodriguez et al., 2025). Meanwhile, the critical thinking cultivated in digital practice may also improve students' problem-solving ability, which corresponds to stronger volitional performance (George et al., 2024).

In column (3), digital literacy was not statistically associated with self-awareness. This result suggests that the improvement of digital literacy may not be directly reflected in the level of self-awareness. Digital literacy is more oriented to the operation and processing of external information and tools, while self-awareness depends more on introspection and self-reflection, and there may be no direct linear association between the two (George et al., 2024).

In column (4), digital literacy was positively associated with personality integration. This pattern aligns with the theoretical expectation that critical information processing helps students form stable value judgment standards, while digital social norm competence and privacy protection awareness support consistent identity performance across online and offline scenarios, which corresponds to a higher level of personality integration (Marchenko and Murzina, 2022).

In column (5), digital literacy was positively associated with interpersonal relationships. This result is consistent with the expectation that digital competence can expand the scope of social interaction and may improve the efficiency of online collaboration and communication (Marchenko and Murzina, 2022). Students with higher digital literacy may perform better in online group work and cross-space interpersonal interaction, which corresponds to better interpersonal relationship quality and collaborative efficacy (Gebremariam et al., 2024).

In column (6), digital literacy was positively associated with adaptability. One possible explanation is that digital literacy, as a general transferable skill, can help students adapt to new learning modes and environmental changes more quickly (Puckett, 2022). Mastery of digital tools and information acquisition and processing can reduce the adjustment demands associated with new learning environments and unfamiliar situations.

All five significant associations survive both wild-cluster bootstrap and FDR correction; self-awareness does not.

### 4.4 Exploratory heterogeneity and non-linearity analyses

Heterogeneity was examined using full-sample interaction models for household registration, gender, living expenses, major, and city type, together with quadratic and spline specifications for potential non-linearity in digital literacy. Monthly living-expense level was retained as a continuous moderator, while household registration, gender, major, and city type were entered according to their prespecified coding. Continuous variables were mean-centered before interaction and polynomial terms were constructed so that lower-order coefficients retain meaningful interpretations. Non-linearity in digital literacy was examined using a quadratic specification rather than by dividing respondents into high- and low-literacy groups. Because all heterogeneity and functional-form analyses are exploratory, inference is based on small-cluster-robust procedures and multiplicity adjustment, and no conclusions are drawn from subgroup-specific significance alone. We report wild-cluster bootstrap *p*-values (Appendix Table E) and BH-FDR-adjusted *p*-values (Appendix Table F) alongside the conventional clustered *p*.

The living-expense interaction provided the strongest suggestive evidence of heterogeneity (β = 0.054; wild-cluster bootstrap *p* = 0.009; BH-FDR adjusted *q* = 0.054). However, *q* = 0.054 narrowly exceeds the 5% FDR threshold. Interactions involving household registration, gender, major, and city type were not robustly distinguishable from zero. The quadratic digital-literacy term was also not significant under wild-cluster inference (*p* = 0.115). No interaction or non-linear specification therefore met the prespecified 5% threshold under both small-cluster and multiplicity-adjusted inference; the findings provide at most exploratory evidence of heterogeneity and do not establish discrete subgroup or threshold effects.

In addition to testing moderation through continuous interaction terms, we directly examined the functional form of the digital-literacy-positive-psychological-functioning association by adding a quadratic term of digital literacy and fitting a one-knot linear spline. As shown in Appendix Table D, the quadratic term is marginally negative and the spline suggests a steeper slope below the sample mean (78.6) that flattens above it; however, neither specification reaches the 5% significance threshold after multiple-testing correction, so we treat this as tentative evidence of non-linearity rather than a confirmed threshold effect.

To address the concern that multiple parallel tests inflate the chance of false positives, we applied the Benjamini-Hochberg false-discovery-rate procedure separately within three test families: the five dimension tests (Table 6), the six sub-dimension tests (Table 7), and the six interaction tests (Table 8). The adjusted *p*-values are reported in Appendix Table F. After correction, digital innovation, the city-type interaction, and the quadratic digital-literacy term do not meet the 5% threshold and are therefore treated as non-robust exploratory patterns.

**Table 7:** Associations between digital literacy and positive psychological functioning dimensions.

Variable	Emotional health	Volitional functioning	Self-awareness	Personality integration	Interpersonal relationships	Adaptability
	(1)	(2)	(3)	(4)	(5)	(6)
Digital literacy	0.059^***^	0.036^**^	0.036	0.019^**^	0.074^***^	0.039^***^
	(5.69)	(2.19)	(1.32)	(2.49)	(3.98)	(4.09)
Wild-bootstrap *p*	0.001	0.041	0.203	0.022	0.006	0.005
BH-FDR *q*	0.006	0.049	0.203	0.033	0.015	0.015
Control variables	YES	YES	YES	YES	YES	YES
*N*	893	893	893	893	893	893
*R* ^2^	0.045	0.025	0.015	0.022	0.038	0.030

Values in parentheses are *t*-statistics based on university-clustered standard errors; ^**^p < 0.05, ^***^p < 0.01. Sub-dimension scores are transformed to a 0–100 scale. Wild-cluster bootstrap *p*-values are based on Webb six-point weights with 1,000 replications. BH-FDR *q*-values are adjusted across the six sub-dimension tests (Appendix Table F).

**Table 8:** Formal heterogeneity tests using continuous-variable interaction terms.

Moderator	Main effect of DL (β)	β	*t*	*p*	Wild-boot *p*	BH-FDR *q*	*R* ^2^
	(1)	(2)	(3)	(4)	(5)	(6)	(7)
Living expenses (continuous)	0.112^***^	0.054^***^	3.21	0.004	0.009	0.054	0.038
Household registration (urban = 1)	0.165^***^	0.021	0.76	0.458	0.471	0.565	0.031
Gender (male = 1)	0.181^***^	−0.018	−0.53	0.603	0.612	0.612	0.031
Major (HSS = 1)	0.183^***^	−0.025	−1.04	0.317	0.335	0.503	0.032
City type (provincial capital = 1)	0.192^***^	−0.041^*^	−1.83	0.089	0.108	0.324	0.033
Digital literacy quadratic term (DL^2^)	0.204^**^	−0.001^*^	−1.76	0.082	0.115	0.324	0.034

Each row adds the indicated interaction to the full adjusted OLS model. The β, *t*, and *p*-values in columns (2)–(4) correspond to the interaction terms. ^***^, ^**^, and ^*^ denote *p* < 0.01, *p* < 0.05, and *p* < 0.10, respectively.

### 4.5 Pathway decomposition: digital information regulation behavior, online social boundary practice, and structured digital use practice

To characterize how the observed cross-sectional association is distributed across three behaviorally proximate constructs, we estimate separate associational decomposition models for digital information regulation behavior, online social boundary practice, and structured digital use practice. These analyses are descriptive and exploratory. Because the decomposition constructs overlap conceptually and empirically with digital literacy and temporal ordering cannot be established, the indirect components are not interpreted as causal mediation effects or additive causal shares.

Because all secondary analyses rely on the same 18 university clusters, we extended the wild-cluster bootstrap correction to every dimension-specific, sub-dimension, interaction, and pathway test. Wild-cluster bootstrap *p*-values for the pathway tests are reported in Appendix Table E; bias-corrected confidence intervals for the indirect components are obtained separately from 2,000 university-level cluster-resampling bootstrap replications. In brief, information acquisition and social interaction remain significant after this correction; tool use, security, and digital innovation do not; and the three indirect pathway effects remain distinguishable from zero.

The first decompositional pathway concerns digital information regulation behavior. As shown in columns (1) and (2), digital literacy is positively associated with digital information regulation behavior, and greater digital information regulation behavior is in turn associated with better positive psychological functioning when digital literacy and controls are included. This pattern is consistent with exploratory decomposition expectation in Exploratory Expectation E1. Students with stronger digital literacy are more likely to adopt structured information regulation behaviors in daily digital use when navigating the volume of online information, enabling them to filter valuable content and resist false or negatively framed information (Raihan et al., 2025). This pattern is consistent with the interpretation that stronger information-regulation practices correspond to less cognitive overload and information anxiety, which in turn is linked to better psychological functioning. However, part of this indirect estimate may stem from measurement redundancy.

The second decomposition component concerns online social boundary practice. Columns (3) and (4) indicate that digital literacy was positively associated with stronger online social boundary practice, which is positively associated with positive psychological functioning. This pattern is consistent with Exploratory Expectation E2 in exploratory decomposition. Digital literacy expands students' access to social opportunities and collaborative platforms, and more importantly, it equips them to manage these interactions strategically. Students with higher digital literacy are better able to coordinate group work, adapt self-presentation across platforms, and protect privacy boundaries. These management practices are associated with greater access to social support and higher emotional satisfaction from digital social contact, which may account for part of the observed cross-sectional association (Tsai et al., 2019). Again, conceptual overlap cannot be ruled out.

The third decomposition component concerns structured digital use practice. Columns (5) and (6) indicate that digital literacy was positively associated with structured digital use practice, and that structured digital use practice was in turn positively associated with positive psychological functioning when digital literacy and controls were included. This pattern is consistent with Exploratory Expectation E3. Students with stronger digital literacy are more likely to plan digital tasks, archive learning materials, and regulate screen time, and these organized digital routines are associated with greater perceived behavioral control, lower digital disorganization, and more adaptive coping (Rahma et al., 2025). The indirect component through structured digital use practice was 0.120 (95% bias-corrected CI [0.069, 0.187]), which excludes zero. Again, because temporal ordering cannot be established and the decomposition construct overlaps empirically and conceptually with digital literacy, this result is interpreted as exploratory associational decomposition rather than as evidence of a causal mechanism, and part of the estimate may reflect shared measurement content.

Bias-corrected confidence intervals for the indirect components were estimated using 2,000 cluster-bootstrap replications, with universities resampled as clusters. The indirect component through digital information regulation behavior was 0.150 (95% CI [0.082, 0.231]); through online social boundary practice, 0.115 (95% CI [0.064, 0.179]); and through structured digital use practice, 0.120 (95% CI [0.069, 0.187]). All three confidence intervals excluded zero, indicating statistically detectable indirect components through digital information regulation behavior, online social boundary practice, and structured digital use practice. Nevertheless, because the three pathways were estimated in separate models and the pathway constructs partially overlap with the overall digital-literacy index, the magnitudes should not be interpreted as additive or causal shares of the total association.

Although all three indirect components were statistically distinguishable from zero under the reported cluster-bootstrap procedure, their magnitudes should not be interpreted as proportions of a causal total effect. Rather, the results indicate that digital literacy, related digital practices, and psychological functioning covary in theoretically meaningful ways in the present cross-sectional sample. Part of the statistical decomposition may reflect shared measurement content.

## 5 Discussion

### 5.1 Principal findings

The observational association is statistically significant yet modest in substantive magnitude: a 10-point increase on the rescaled 0–100 digital-literacy index corresponds to a 1.78-point increase on the 0–100 positive psychological-functioning composite; the adjusted *R*^2^ of 0.031 indicates that the model explains only a small share of the variance in positive psychological functioning.

The positive-psychological functioning dimension-level analysis further indicates that the overall association was concentrated in emotional health, volitional functioning, personality integration, interpersonal relationships, and adaptability, with no statistically significant association for self-awareness. This pattern suggests that digital literacy may be more closely related to students' functioning in tasks, relationships, and changing environments than to reflective self-understanding. Formal continuous-variable interaction tests indicated statistically detectable heterogeneity only by living-expense level, with marginal evidence for city type and tentative non-linear pattern for digital literacy. The apparent differences by household registration, gender, and major were not statistically significant. The subgroup findings should therefore be interpreted as exploratory.

### 5.2 Interpretation of unexpected findings

The negative association for information acquisition and processing may have several explanations. First, the measure may partly capture the intensity of information engagement rather than the efficiency of information regulation. Students who search frequently and integrate information from multiple sources may be more exposed to conflicting content, uncertainty, and cognitive overload. Second, reverse causality is possible: students experiencing distress may search for more information in an effort to reduce uncertainty. Third, if the scale combines retrieval skill, risk awareness, and information exposure, its coefficient may reflect heterogeneous processes that operate in opposite directions. The result should therefore be interpreted as an association that warrants further measurement refinement, not as evidence that information-processing competence harms psychological functioning.

The negative association for digital innovation may reflect the demands accompanying creative digital work. Programming, content creation, and cross-domain innovation often require sustained attention, repeated trial and error, public performance, and continuous skill updating. These demands may increase time pressure and self-evaluative stress, especially when innovation is linked to academic competition or employment preparation. An alternative explanation is selection: students facing greater academic or career pressure may engage more intensively in digital innovation. Longitudinal data are needed to distinguish these possibilities.

The non-significant association for digital security and ethical awareness may indicate that this dimension is more distal from everyday psychological functioning than practical tool use or social interaction. Security knowledge may have a protective value mainly when students encounter actual privacy breaches, fraud, cyberbullying, or other online risks. A ceiling effect or limited variation in self-reported security awareness could also reduce statistical power. Future studies should measure both security knowledge and actual exposure to digital risks and test whether risk exposure moderates the association.

### 5.3 Theoretical implications

The findings support a capability-resource-regulation perspective in which digital literacy is related to positive psychological functioning through several proximal processes rather than through a single uniform mechanism. The results also show why the five dimensions should be mapped explicitly onto cognitive, relational, and behavioral pathways. However, the conceptual proximity between the digital-literacy dimensions and the decomposition constructs remains a limitation. The pathway results are best viewed as preliminary exploratory associational evidence that specific enactments of digital competence may account for part of the overall cross-sectional association. Stronger tests would use non-overlapping indicators, latent-variable models, and longitudinal measurement.

### 5.4 Methodological interpretation

Several features constrain causal interpretation. The cross-sectional design does not establish temporal order, all primary variables are self-reported, and common-method variance may inflate associations. Small-cluster bias risk exists given only 18 university clusters, and we rely on wild-cluster bootstrap to mitigate this concern. In addition, city-level internet penetration may be related to psychological functioning through pathways other than digital literacy, so the instrumental-variable exclusion restriction cannot be verified. Accordingly, consistency across models increases confidence in the robustness of the observed association but does not establish a causal effect. The subgroup and pathway-decomposition findings should also be considered exploratory until replicated with prospective data and stronger measurement separation. FDR adjustment for multiple comparisons is applied for exploratory tests; several suggestive dimension-specific and subgroup patterns lose significance after multiplicity correction.

## 6 Conclusions and implications

### 6.1 Conclusions

Using cross-sectional survey data from 893 college students across 18 universities in Henan Province, this study found that overall digital literacy was positively associated with positive psychological functioning, conceptualized as psychological functioning and adaptive adjustment. The magnitude of this association was modest: a 10-point increase in digital literacy corresponded to approximately a 1.78-point higher score on the 0–100 positive-psychological-functioning composite, and the adjusted model explained a limited proportion of outcome variance.

The most consequential theoretical finding is that the association between digital literacy and positive-psychological functioning is internally heterogeneous rather than uniformly positive. Dimension-specific analyses indicated that the association was not uniform across components of digital literacy. After accounting for small-cluster inference and multiple testing, information acquisition and processing remained negatively associated with positive psychological functioning, whereas digital social interaction remained positively associated. Digital tool use proficiency, digital innovation, and digital security and ethical awareness did not show robust associations under the corrected inference procedures. We note, however, that the negative coefficient for digital innovation did not survive small-cluster and multiplicity-adjusted inference, so the interpretation above should be read as hypothesis-generating rather than as a confirmed finding. Across positive-psychological-functioning dimensions, digital literacy was positively associated with emotional health, volitional functioning, personality integration, interpersonal relationships, and adaptability, whereas the association with self-awareness was not statistically detectable.

Exploratory heterogeneity and non-linearity analyses did not provide robust evidence of subgroup moderation or non-linear functional form after conservative correction, although the living-expense interaction showed suggestive evidence warranting replication. The associational decomposition analyses identified statistically detectable indirect components through digital information regulation behavior, online social boundary practice, and structured digital use practice. Because these constructs overlap conceptually and empirically with digital literacy and the data are cross-sectional, the decomposition results do not establish causal mediation.

Taken together, the findings support a multidimensional and appropriately cautious understanding of digital literacy as one correlate of college students' psychological functioning. Digital-literacy education may have educational value in supporting purposeful information management, constructive online interaction, and balanced digital engagement, but the present evidence does not establish that digital-literacy interventions will produce substantial improvements in positive psychological functioning.

### 6.2 Limitations and future research

This study has several limitations that should be acknowledged. First, the data are cross-sectional, so temporal ordering, reverse causality, and causal mediation cannot be established. Longitudinal and experimental studies are needed to examine whether changes in digital literacy precede changes in positive psychological functioning.

Second, the measures are based on self-reports and may be affected by social desirability, recall error, and common-method variance. Future research should combine questionnaires with behavioral indicators, such as verified learning-platform use, information-search patterns, or digital wellbeing measures, while protecting participants' privacy. Split-sample EFA-CFA is implemented in current revision, yet several AVE values remain below 0.50. Future work should further refine item pools to achieve better convergent and discriminant validity, especially for pathway constructs.

Third, the sample is a convenience, online-recruited sample rather than a probability-based sample of college students in Henan Province. The 18 participating universities were those represented in the final non-probability online-recruited sample; they were not selected through a probability-based institutional sampling frame, and students were self-selected through Questionnaire Star rather than randomly drawn from institutional rosters. Institutions with stronger survey circulation or student participation may be overrepresented, and the findings should not be interpreted as representative of college students in Henan Province more broadly or of Chinese college students nationally. Multi-province, probability-based studies with institutional rosters are needed to confirm the present associations.

Relatedly, both the digital-literacy and positive psychological functioning measures were study-specific instruments rather than established standardized scales. Although expert review, pilot testing, reliability analysis, and split-sample factor analysis provided preliminary psychometric support, further independent validation is needed before these composites can be used as standardized measures in other samples.

Fourth, several pathway items are conceptually close to dimensions of the digital-literacy scale, and the three-item pathway scales may be sensitive to measurement error with relatively low internal consistency for some scales. Future studies should develop non-overlapping multi-item measures and evaluate them using confirmatory factor analysis and longitudinal mediation models.

Fifth, city-level internet penetration may affect psychological functioning through channels other than digital literacy; the instrumental-variable results should therefore be treated only as supplementary sensitivity evidence, not as definitive causal identification.

Sixth, the study conducts multiple exploratory dimension-specific and subgroup analyses, which may increase false-positive risk. We apply Benjamini-Hochberg FDR adjustment to address multiplicity. In addition, with only 18 university clusters, conventional clustered standard errors may be affected by small-cluster bias; wild-cluster bootstrap results are provided as an additional check but cannot fully resolve this concern. Future research should pre-specify heterogeneity hypotheses, use formal continuous-variable interaction or non-linear tests, and consider adjustments for multiple comparisons.

### 6.3 Practical implications

The findings suggest several cautious educational considerations. Because the evidence is observational and cross-sectional, it does not establish that digital-literacy interventions improve psychological functioning, and the observed association is modest.

First, universities may consider integrating basic digital tool use with healthy online interaction rather than treating digital literacy as a purely technical curriculum. Short, task-based modules can focus on academic software, collaborative platforms, communication boundaries, and the use of online support resources. Although the coefficient for digital tool use proficiency was positive, it was not statistically significant. Practical digital-tool training is therefore recommended primarily for its intrinsic educational value rather than as an empirically confirmed psychological-functioning intervention. Any mental-health-related benefit is likely limited.

The negative association for information acquisition and processing, which remained statistically detectable after the reported corrections, warrants attention to information-load regulation and source evaluation in future research and educational design; it should not be interpreted as evidence that information-processing competence is harmful. The negative coefficient for digital innovation was not robust after small-cluster and multiplicity corrections and therefore should not be used as an empirical basis for intervention design.

Third, from an equity perspective, universities may prioritize students with limited access to digital resources, including some students from rural backgrounds or institutions outside the provincial capital. This recommendation is based on considerations of resource accessibility rather than on statistically established subgroup differences in the present study. The suggestive living-expense interaction and tentative non-linear pattern warrant further investigation before being translated into targeted interventions. The living-expense interaction was statistically significant under wild-cluster bootstrap inference alone (*p* = 0.009) but narrowly failed the 5% BH-FDR threshold (*q* = 0.054).

## Data Availability

The raw data supporting the conclusions of this article will be made available by the authors, without undue reservation.
