# Supplementary material for: Digital literacy and positive psychological functioning among college students in the digital era: evidence from a multi-university sample in Henan Province, China
Source: Front Psychol. 2026 Oct 29;17:1966399. doi: 10.3389/fpsyg.2026.1966399 (PMC13755188; doi:10.3389/fpsyg.2026.1966399)
Supplement: Supplementary file 1 [file Table_1.docx]

**Appendix**

**Appendix A. Split-Sample EFA (Calibration n=446) and CFA (Validation n=447): Factor Loadings**

Selected primary and secondary pattern coefficients from the split-sample EFA, together with standardized CFA loadings and model-fit indices, are reported in Appendix Table A. We randomly split the full sample into a calibration subsample (n=446) for exploratory factor analysis and a validation subsample (n=447) for confirmatory factor analysis, to avoid over-fitting the factor structure to the same data used to evaluate it. The EFA recovers the intended five digital-literacy factors and the six positive-psychological-functioning factors (with personality, interpersonal, and adaptability collapsing into one broad factor on the EFA side, which CFA then separates). Standardized CFA loadings are mostly between 0.66 and 0.84, and the overall fit is acceptable (χ²/df=2.31, CFI=0.91, RMSEA=0.054). We acknowledge transparently that two AVE values among the digital-literacy dimensions fall below 0.50 and that convergent validity is weaker for several other constructs, particularly the pathway constructs.

Appendix Table A. Split-Sample Measurement Validation: EFA Pattern Coefficients and CFA Standardized Loadings

| Item | F1 Tool | F2 Info | F3 Social | F4 Innov. | F5 Security | F6 Emot. | F7 Volit. | F8 Self | F9 Other* | EFA loading | CFA std. loading |
| --- | --- | --- | --- | --- | --- | --- | --- | --- | --- | --- | --- |
| DL1 Tool operation | 0.71 | 0.12 | 0.08 | 0.05 | 0.1 | — | — | — | — | 0.74 | 0.82 |
| DL2 Problem-solving | 0.68 | 0.15 | 0.11 | 0.09 | 0.06 | — | — | — | — | 0.71 | 0.79 |
| DL3 Keyword retrieval | 0.08 | 0.74 | 0.09 | 0.12 | 0.05 | — | — | — | — | 0.76 | 0.83 |
| DL4 Information discrimination | 0.11 | 0.71 | 0.13 | 0.06 | 0.15 | — | — | — | — | 0.73 | 0.8 |
| DL5 Multi-source integration | 0.14 | 0.69 | 0.08 | 0.15 | 0.09 | — | — | — | — | 0.7 | 0.77 |
| DL6 Online collaboration | 0.06 | 0.1 | 0.76 | 0.12 | 0.08 | — | — | — | — | 0.78 | 0.84 |
| DL7 Online etiquette | 0.09 | 0.08 | 0.72 | 0.05 | 0.13 | — | — | — | — | 0.72 | 0.79 |
| DL8 Risk prevention | 0.13 | 0.11 | 0.68 | 0.09 | 0.16 | — | — | — | — | 0.69 | 0.76 |
| DL9 Digital creation | 0.07 | 0.12 | 0.1 | 0.73 | 0.06 | — | — | — | — | 0.75 | 0.81 |
| DL10 Cross-domain integration | 0.1 | 0.09 | 0.14 | 0.7 | 0.11 | — | — | — | — | 0.71 | 0.78 |
| DL11 Privacy management | 0.12 | 0.07 | 0.11 | 0.08 | 0.72 | — | — | — | — | 0.74 | 0.8 |
| DL12 Ethical judgment | 0.06 | 0.1 | 0.15 | 0.12 | 0.69 | — | — | — | — | 0.7 | 0.77 |
| EH1 Emotional stability (R) | — | — | — | — | — | 0.72 | — | — | — | 0.73 | 0.8 |
| EH2 Positive attitude | — | — | — | — | — | 0.75 | — | — | — | 0.77 | 0.83 |
| EH3 Emotion regulation | — | — | — | — | — | 0.68 | — | — | — | 0.69 | 0.76 |
| VF1 Career planning | — | — | — | — | — | — | 0.74 | — | — | 0.75 | 0.81 |
| VF2 Resilience | — | — | — | — | — | — | 0.71 | — | — | 0.72 | 0.79 |
| VF3 Goal execution | — | — | — | — | — | — | 0.69 | — | — | 0.7 | 0.77 |
| SA1 Strengths/weaknesses | — | — | — | — | — | — | — | 0.73 | — | 0.74 | 0.8 |
| SA2 Accept feedback | — | — | — | — | — | — | — | 0.7 | — | 0.71 | 0.78 |
| SA3 Role matching | — | — | — | — | — | — | — | 0.66 | — | 0.67 | 0.74 |
| PI1 Value consistency | — | — | — | — | — | — | — | — | 0.72 | 0.73 | 0.8 |
| PI2 Situational consistency | — | — | — | — | — | — | — | — | 0.7 | 0.71 | 0.78 |
| PI3 Cognition-emotion-behavior | — | — | — | — | — | — | — | — | 0.68 | 0.69 | 0.76 |
| IR1 Conflict resolution | — | — | — | — | — | — | — | — | 0.71 | 0.72 | 0.79 |
| IR2 Empathy | — | — | — | — | — | — | — | — | 0.74 | 0.75 | 0.82 |
| IR3 Team contribution | — | — | — | — | — | — | — | — | 0.69 | 0.7 | 0.77 |
| AD1 Environment adaptation | — | — | — | — | — | — | — | — | 0.67 | 0.68 | 0.75 |
| AD2 Learning adaptation | — | — | — | — | — | — | — | — | 0.7 | 0.71 | 0.78 |
| AD3 Cultural tolerance | — | — | — | — | — | — | — | — | 0.65 | 0.66 | 0.73 |

Note: F9 bundles personality, interpersonal, and adaptability items in the EFA; the CFA separates them into three correlated factors (r between 0.42 and 0.58). EFA used principal axis factoring with promax rotation. KMO=0.847; Bartlett χ²(741)=8926.3, p<0.001. CFA fit: χ²/df=2.31, CFI=0.91, TLI=0.90, RMSEA=0.054, SRMR=0.061. AVE: digital literacy dimensions 0.46–0.58 (two below 0.50); Positive psychological functioning dimensions 0.44-0.55; pathway constructs 0.39–0.46. Discriminant validity is partial; squared inter-construct correlations exceed AVE for two pairs involving pathway constructs.

**Appendix B. Wild-Cluster Bootstrap Baseline Check**

With only 18 university clusters, the conventional cluster-robust standard error can be biased downward. We therefore re-estimate the statistical significance of the three baseline digital-literacy coefficients (OLS, factor-score index, entropy-weighted index) using the wild-cluster bootstrap with Webb's six-point weight distribution and 1000 replications, which is the recommended approach when the number of clusters is small. As shown in Appendix Table B, all three baseline coefficients remain significant at the 1% level.

Across the three baseline models, the wild-bootstrap p-values remain below 0.001, so the headline association between digital literacy and positive psychological functioning is robust to small-cluster inference. Instrumental‑variable sensitivity analysis using city‑level internet penetration is reported in Appendix Table C. Due to untestable exclusion restriction and weak‑identification risk, IV analysis serves only as supplementary sensitivity check and cannot establish causal effects.

Appendix Table B Wild-Cluster Bootstrap Sensitivity for Baseline Models

| Model | Coefficient | Clustered SE | Clustered p | Wild Bootstrap p |
| --- | --- | --- | --- | --- |
| Baseline OLS (Table 4, col. 2) | 0.178 | 0.035 | <0.001 | <0.001 |
| Factor-score index | 0.162 | 0.033 | <0.001 | <0.001 |
| Entropy-weighted index | 0.169 | 0.034 | <0.001 | <0.001 |

Note: Webb six-point distribution used because only 18 university clusters are available.

**Appendix C. Instrumental-Variable Sensitivity Analysis**

We move the IV analysis to the appendix and present it strictly as a supplementary sensitivity check. We use city-level internet penetration as the instrument for individual digital literacy, estimate the model by two-stage least squares, and report the first-stage and second-stage results together with the weak-instrument diagnostic (Kleibergen–Paap F) and the endogeneity test.

The first stage shows that city-level internet penetration is positively associated with individual digital literacy. The first-stage Kleibergen-Paap rk Wald F statistic was 12.89. Although this exceeds the conventional rule-of-thumb value of 10, it does not eliminate weak-identification concerns, particularly given the limited number of clusters. The second-stage coefficient (β=0.184) is close to the OLS estimate. We do not treat this as causal evidence: city-level internet penetration likely correlates with local economic development, employment opportunities, and the availability of counseling services, all of which could independently affect mental health through channels other than digital literacy. The IV estimate is reported solely as a supplementary sensitivity analysis and is not used to strengthen causal interpretation of the OLS association.

Appendix Table C. Instrumental-Variable Sensitivity Analysis (Supplementary Only)

| First stage (1) | Second stage (2) |
| --- | --- |
| 2.315^***^ |  |
| (3.59) |  |
|  | 0.184^***^ |
|  | (5.51) |
| 12.89 |  |
| 0.003 |  |
| 893 | 893 |
| 0.387 | 0.032 |

Note: City-level internet penetration is plausibly related to local economic development, employment, digital-resource availability, and access to health/counseling services; the exclusion restriction is therefore not defensible. Kleibergen–Paap F=12.89 indicates a borderline instrument. These results are a supplementary sensitivity check only and do not establish causal effects.

**Appendix D. Nonlinearity Tests**

Instead of splitting students into "low" and "high" digital-literacy groups at the median, we test functional form directly. We first add a quadratic term (digital literacy squared) to the baseline model; a statistically detectable quadratic term provides evidence of curvature within the specified quadratic functional form. We then fit a simple linear spline with one knot placed at the sample mean (78.6), allowing the slope to differ below and above the mean. Both specifications include the full control set. The specific results are shown in Appendix Table D.

The quadratic term was negative under conventional clustered inference (p=0.082) but was not significant under wild-cluster bootstrap (p=0.115) or BH-FDR correction (q=0.324). The spline shows a steeper positive slope below the mean (β=0.221) that flattens above it (β=0.084), but the difference across the knot was not robust to small-cluster or multiplicity correction. We interpret this as tentative evidence that the association may be stronger at lower levels of digital literacy and weaker once students are already competent, but because the pattern does not survive FDR correction, we do not build substantive conclusions on it.

Appendix Table D. Nonlinearity Tests: Quadratic Term and Linear Spline

| Specification | β | SE | t | p | R² |
| --- | --- | --- | --- | --- | --- |
| (1) Linear DL only (baseline) | 0.178^***^ | 0.035 | 5.14 | <0.001 | 0.031 |
| (2) DL linear term | 0.204^**^ | 0.092 | 2.22 | 0.041 | 0.033 |
| DL² (quadratic) | -0.001^*^ | 0.001 | -1.76 | 0.082 |  |
| (3) Spline–slope below knot (mean=78.6) | 0.221^***^ | 0.051 | 4.33 | <0.001 | 0.034 |
| (4) Spline–slope above knot | 0.084 | 0.049 | 1.71 | 0.106 |  |
| (5) Difference across spline knot | -0.137^*^ | 0.071 | -1.93 | 0.072 |  |

Note: Knot placed at sample mean of digital literacy (78.63). All models include full controls. The quadratic term is marginally negative; the spline suggests a steeper positive slope below the mean and a flatter slope above. This is interpreted as tentative nonlinearity, not as evidence for a high/low subgroup moderator.

**Appendix E. Wild-Cluster Bootstrap p-Values for Secondary Analyses**

For the sake of robustness in regression results, we extend small-cluster correction beyond the baseline model to all dimension-specific, sub-dimension, interaction, and pathway tests. We therefore re-estimate p-values under the wild-cluster bootstrap for every secondary regression reported in Tables 5, 6, 7, and 8, using the same Webb six-point distribution and 1,000 replications clustered at the university level. As shown in Appendix Table E, dimension-level, sub-dimension-level, interaction, and pathway p-values are re-estimated under this correction.

In Appendix Table E, at the digital-literacy dimension level, information acquisition and social interaction remain significant; tool use and security remain non-significant. Digital innovation, which was marginal under the conventional test, moves to p=0.116 and is no longer described as marginally significant. At the positive-psychological-functioning sub-dimension level, five of the six associations (emotional, volitional, personality, interpersonal, adaptability) remain significant after bootstrap; self-awareness remains non-significant. Among the interaction tests, only the living-expense interaction remained statistically significant under wild-cluster bootstrap inference alone (p=0.009); however, it did not survive BH-FDR adjustment (q=0.054); city type and the quadratic term move to non-significant after bootstrap correction. The three indirect pathway effects remain distinguishable from zero. In the main text, all conclusions have been downgraded to match this more conservative inference.

Appendix Table E. Wild-Cluster Bootstrap p-Values for Secondary Analyses

| Test | Original clustered p | Wild-bootstrap p (1000 reps) | Inference under wild bootstrap |
| --- | --- | --- | --- |
| Sub-dimension: tool use | 0.102 | 0.148 | ns |
| Sub-dimension: info acquisition | <0.001 | 0.003 | significant |
| Sub-dimension: social interaction | <0.001 | 0.001 | significant |
| Sub-dimension: innovation | 0.074 | 0.116 | ns (was marginal) |
| Sub-dimension: security | 0.294 | 0.347 | ns |
| Sub-dimension: emotional health | <0.001 | 0.001 | significant |
| Sub-dimension: volitional functioning | 0.035 | 0.041 | significant |
| Sub-dimension: self-awareness | 0.187 | 0.203 | ns |
| Sub-dimension: personality integration | 0.021 | 0.022 | significant |
| Sub-dimension: interpersonal relationships | <0.001 | 0.006 | significant |
| Sub-dimension: adaptability | <0.001 | 0.005 | significant |
| Interaction: living expenses | 0.004 | 0.009 | Significant (wild bootstrap only) |
| Interaction: household registration | 0.458 | 0.471 | ns |
| Interaction: gender | 0.603 | 0.612 | ns |
| Interaction: major | 0.317 | 0.335 | ns |
| Interaction: city type | 0.089 | 0.108 | ns (was marginal) |
| Quadratic DL² | 0.082 | 0.115 | ns (was marginal) |
| Indirect: info regulation | <0.001 | <0.001 | CI excludes 0 |
| Indirect: social boundary | <0.001 | <0.001 | CI excludes 0 |
| Indirect: structured use | <0.001 | <0.001 | CI excludes 0 |

Note: Webb six-point distribution, 18 university clusters. Results with wild-cluster bootstrap p ≥ 0.05 are not treated as statistically significant under the small-cluster robustness check. “ns” denotes not statistically significant.

**Appendix F. Benjamini–Hochberg FDR-Adjusted p-Values**

Because multiple hypotheses are tested within each family of dimension, sub-dimension, and interaction analyses, we apply the Benjamini–Hochberg false-discovery-rate procedure separately within each family to control the expected proportion of false discoveries among rejected hypotheses. The resulting adjusted p-values are reported in Appendix Table F.

After FDR adjustment, the core findings survive: information acquisition (negative), social interaction (positive), and the emotional, volitional, personality, interpersonal, and adaptability sub-dimensions. The living-expense interaction, although the strongest among the six heterogeneity tests (wild p=0.009), narrowly missed the 5% BH-FDR threshold (q=0.054) and is therefore treated as suggestive rather than significant. All other tests, including tool use, security, digital innovation, self-awareness, the city-type interaction, the quadratic digital-literacy term, and the household-registration, gender, and major interactions, are treated as non-robust exploratory patterns.

Appendix Table F1: 5 Dimension Tests (Table 5)

| Test | Tool use | Information acquisition | Social interaction | Digital innovation | Security & ethical |
| --- | --- | --- | --- | --- | --- |
| Raw wild p | 0.148 | 0.003 | 0.001 | 0.116 | 0.347 |
| BH-FDR q | 0.193 | 0.008 | 0.005 | 0.193 | 0.347 |
| Significant at 5%? | No | Yes | Yes | No | No |

Appendix Table F2: 6 sub-dimensions (Table 6)

|  | Test | Emotional health | Volitional functioning | Self-awareness | Personality integration | Interpersonal relationships | Adaptability |
| --- | --- | --- | --- | --- | --- | --- | --- |
|  | Raw wild p | 0.001 | 0.041 | 0.203 | 0.022 | 0.006 | 0.005 |
|  | BH-FDR q | 0.006 | 0.049 | 0.203 | 0.033 | 0.015 | 0.015 |
|  | Significant at 5%? | Yes | Yes | No | Yes | Yes | Yes |

Appendix Table F3: 6 interaction tests (Table 7)

| Test | Living expenses | Household registration | Gender | Major | City type | DL² nonlinearity |
| --- | --- | --- | --- | --- | --- | --- |
| Raw wild p | 0.009 | 0.471 | 0.612 | 0.335 | 0.108 | 0.115 |
| BH-FDR q | 0.054 | 0.565 | 0.612 | 0.503 | 0.324 | 0.324 |
| Significant at 5%? | No | No | No | No | No | No |
